# A Deep Learning-Based Quantitative Structure–Activity Relationship System Construct Prediction Model of Agonist and Antagonist with High Performance

**DOI:** 10.3390/ijms23042141

**Published:** 2022-02-15

**Authors:** Yasunari Matsuzaka, Yoshihiro Uesawa

**Affiliations:** 1 Department of Medical Molecular Informatics, Meiji Pharmaceutical University, Kiyose 204-8588, Japan; yasunari80808@ims.u-tokyo.ac.jp; 2 Center for Gene and Cell Therapy, Division of Molecular and Medical Genetics, The Institute of Medical Science, University of Tokyo, Minato City 108-8639, Japan

**Keywords:** cheminformatics, computer aided molecular design, deep learning, molecular remodeling, QSAR

## Abstract

Molecular design and evaluation for drug development and chemical safety assessment have been advanced by quantitative structure–activity relationship (QSAR) using artificial intelligence techniques, such as deep learning (DL). Previously, we have reported the high performance of prediction models molecular initiation events (MIEs) on the adverse toxicological outcome using a DL-based QSAR method, called DeepSnap-DL. This method can extract feature values from images generated on a three-dimensional (3D)-chemical structure as a novel QSAR analytical system. However, there is room for improvement of this system’s time-consumption. Therefore, in this study, we constructed an improved DeepSnap-DL system by combining the processes of generating an image from a 3D-chemical structure, DL using the image as input data, and statistical calculation of prediction-performance. Consequently, we obtained that the three prediction models of agonists or antagonists of MIEs achieved high prediction-performance by optimizing the parameters of DeepSnap, such as the angle used in the depiction of the image of a 3D-chemical structure, data-split, and hyperparameters in DL. The improved DeepSnap-DL system will be a powerful tool for computer-aided molecular design as a novel QSAR system.

## 1. Introduction

Quantitative structure–activity relationship (QSAR) models can reduce the time and cost of molecular screening through mathematical prediction models of regression or classification of properties and activities of a chemical compound based on their chemical structure and statistically significant corresponding physicochemical/toxicological properties with other methods such as homology modeling, molecular docking, and molecular dynamics (MD) simulation [1,2,3,4,5,6,7,8,9,10,11,12,13,14,15,16,17,18,19,20,21,22,23,24,25,26,27,28,29,30,31,32,33,34]. The structure-based molecular design mainly includes a receptor-based method through a three-dimensional (3D) chemical structure to obtain ligand interaction [1,35,36]. However, traditional QSAR models may frequently miss suitable candidate molecules, because of the poor predictive accuracy and versatility caused by poor feature selection that requires skill and knowledge and conformational limitations for coincidence effect [1,37,38,39]. Therefore, a QSAR system with high-throughput and performance is desired because of the development of novel medicines, chemicals, and nanomaterials on human health. The important factor for solving the QSAR issue is the extraction of information-rich numerical molecular descriptors associated with physicochemical/toxicological properties. However, 3D-QSAR has a high computational cost, and its performance is sensitive to changes in the ligand geometry such as conformation and orientation [1,40]. To resolve these drawbacks, 4D-QSAR, called MD-QSAR, applied the ligand geometry problem for the effective ligand constrains using a 4D-chemical descriptor with multiple structural conformation, orientation, and protonation state calculated through short run MD stimulation to approximate the Boltzman sampling [1,41,42]. Although the 4D-QSAR can reduce the bias by selecting conformation, orientation, and protonation state, it requires more adaptation of the ligand topology within its target protein-binding pocket [43]. Thus, 5D-QSAR was proposed explicitly to represent different induced-fit models in 4D-QSAR [1,41,43,44,45]. Furthermore, 6D-QSAR was introduced incorporating different solvation models in 5D-QSAR [1,41,46].

A DL-based QSAR system, called DeepSnap-DL, was reported to capture molecular features from molecular images photographed on a 3D-chemical structure [47]. In the DeepSnap-DL system, parameters for depicting a ball-and-stick model of chemical structure influenced prediction-performances of toxicity activity of the molecular initiation event (MIE) molecule, which interacts with protein and/or DNA in an adverse outcome pathway induced by chemical compounds in human body using the Tox21 10k library, including about 10,000 (10k) chemicals, e.g., approved drugs and environmental chemicals [48,49,50]. The prediction models using the DeepSnap-DL system achieved higher performance than conventional ML techniques, such as random forest, XGBoost, LightGBM, and CatBoost [51,52]. Additionally, prediction models of MIE molecule agonist or antagonist activity were constructed using by the DeepSnap-DL system with the Tox21 10k library, suggesting this system as essential tool for novel QSAR analysis due to automatic feature extraction with numerous structural information from a 3D-chemical structure [53,54]. For high-throughput of the DeepSnap-DL system, automation in the DeepSanp-DL system has been conducted by combining each process consisting of the generation of images from a 3D-chemical structure based on the simplified molecular input line entry system (SMILES) format, DL using these images as input data, and calculation of prediction-performance indexes using TensorFlow and Keras [54]. In the modified DeepSnap-DL system, the mean values of *receiver operating characteristic* area under the curve (ROC_AUC) of the prediction models for 59 MIE targets in validation, test, and foldout datasets indicated 0.818 ± 0.056, 0.803 ± 0.063, and 0.792 ± 0.076, respectively [54]. Furthermore, two of the MIE targets, peroxisome proliferator activated receptor γ (PPARγ) agonist (PPARg_ago, AID:743140) and aromatase antagonist (Arom_ant, AID:743139), improved the prediction-performance by optimizing of parameters in the modified DeepSnap-DL system, such as angle in the depiction of the image from 3D-chemicals, data-split ratio with training (train), validation (valid), and test datasets, background color in an image, and learning rate (LR) and batch size (BS) in hyperparameters in DL [54].

In this study, we used the modified DeepSnap-DL with Python and basic DeepSnap-DL with DIGITS systems to construct prediction models in three of MIEs, glucocorticoid receptor (PubChem assay AID:720725_GR_ant), *transforming growth factor* (TGF)-beta/Smad (PubChem assay AID:1347032_TGF_beta_ant), and thyrotropin-releasing hormone receptor (PubChem assay AID:1347030_TRHR_ago), by optimizing parameters in the DeepSnap-DL system. According to the previously reported MIE molecules, agonist, or antagonist prediction models in the three MIE molecules constructed using the modified DeepSnap-DL with Python showed that it would be essential tools in a novel QSAR system in computer-aided molecular design.

## 2. Results and Discussion

### 2.1. Angles and Data Split in DeepSnap-DL with DIGITS and Python Systems

To analyze the influence of different angles on the snapshot generation of DeepSnap_Python and DeepSnap_DIGITS as 256 × 256 pixel PNG files, we used 31 and 23 from 65°, 65°, 65° to 350°, 350°, 350° in Python and from 70°, 70°, 70° to 345°, 345°, 345° in DIGITS of 720725_GR_ant, 15 and 17 from 95°, 95°, 95° to 325°, 325°, 325° in Python and from 95°, 95°, 95° to 355°, 355°, 355° in DIGITS of 1347030_TRHR_ago, 16 and 16 from 75°, 75°, 75° to 350°, 350°, 350° in Python and from 75°, 75°, 75° to 350°, 350° 350° in DIGITS of 1347032_TGF_beta_ant, different angles (Table 1). Additionally, to examine the influence of different splits among the train, valid, and test datasets, seven types train:valid:test = 1:1:1, 2:2:1, 3:3:1, 4:4:1, 5:5:1, 5:3:2, 7:1:2 in DeepSnap_Python and DeepSnap_DIGITS of 720725_GR_ant, three types train:valid:test = 1:1:1, 3:1:2, 5:3:4, in DeepSnap_Python and DeepSnap_DIGITS of 1259395_TSHR_ant, and eight types train:valid:test = 1:1:1, 2:2:1, 3:1:1, 3:2:1, 5:3:2, 5:5:1, 6:1:2, 7:1:2 in DeepSnap_Python and DeepSnap_DIGITS of 1347032_TGF_beta of data-split ratios were prepared (Table 2).

**Figure 1:**
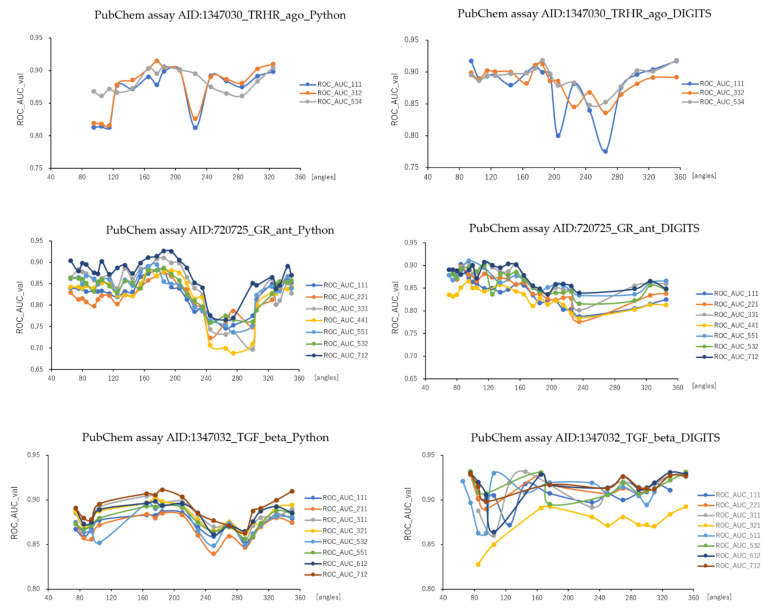
ROC_AUC with angles in DeepSnap-DL using TensorFlow and DIGITS of prediction models of the glucocorticoid receptor (PubChem assay AID:720725_GR_ant), TGF-beta/Smad (PubChem assay AID:1347032_TGF_beta_ant), and agonist of the thyrotropin-releasing hormone receptor (PubChem assay AID:1347030_TRHR_ago) in the validation dataset; *n* = 3.

**Figure 2:**
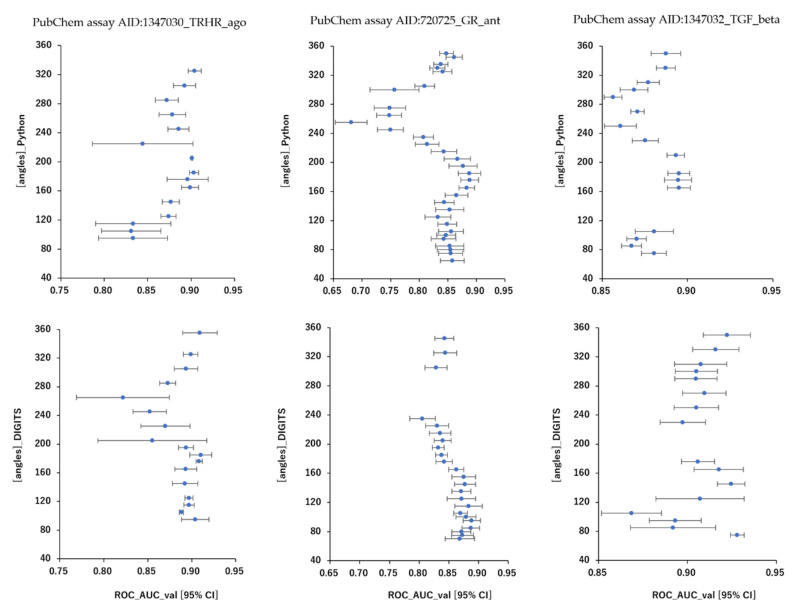
Differences in mean ROC_AUC levels shown as dots with 95% confident interval (95% CI) as error bars with angles in DeepSnap-DL using TensorFlow and DIGITS of prediction models of the glucocorticoid receptor (PubChem assay AID:720725_GR_ant), TGF-beta/Smad (PubChem assay AID:1347032_TGF_beta_ant), and agonist of the thyrotropin-releasing hormone receptor (PubChem assay AID:1347030_TRHR_ago) in the validation dataset; *n* = 3.

**Figure 3:**
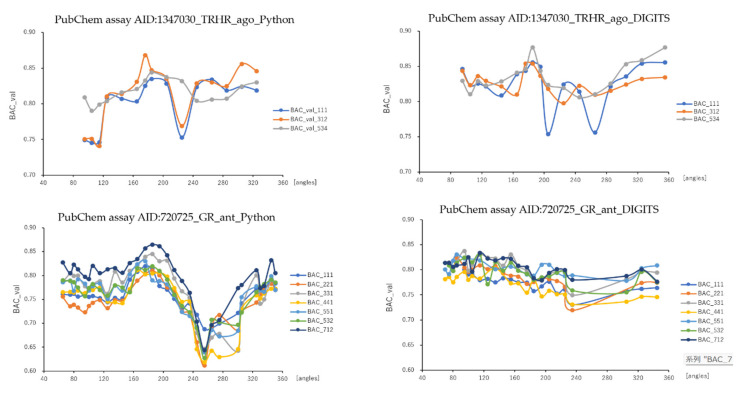

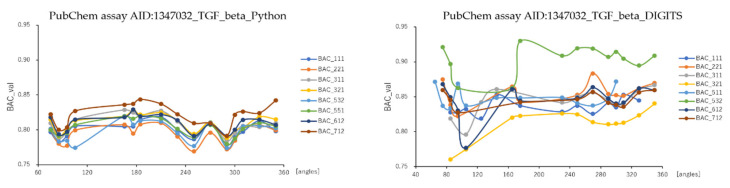
BAC with angles in DeepSnap-DL using TensorFlow and DIGITS of prediction models of the glucocorticoid receptor (PubChem assay AID:720725_GR_ant), TGF-beta/Smad (PubChem assay AID:1347032_TGF_beta_ant), and agonist of the thyrotropin-releasing hormone receptor (PubChem assay AID:1347030_TRHR_ago) in the validation dataset; *n* = 3.

**Figure 4:**
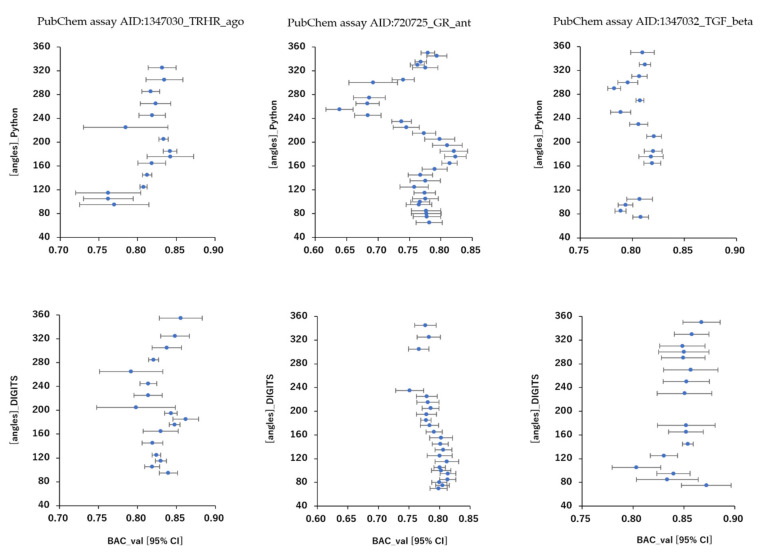
Differences in mean BAC levels shown as dots with 95% confident interval (95% CI) as error bars with angles in DeepSnap-DL using TensorFlow and DIGITS of prediction models of the glucocorticoid receptor (PubChem assay AID:720725_GR_ant), TGF-beta/Smad (PubChem assay AID:1347032_TGF_beta_ant), and agonist of the thyrotropin-releasing hormone receptor (PubChem assay AID:1347030_TRHR_ago) in the validation dataset; *n* = 3.

As results, DeepSnap_Python and DeepSnap_DIGITS in the three MIE targets achieved the following prediction-performance. The mean ROC_AUC, BAC, MCC, and Acc values in the valid dataset were 0.832 ± 0.048 for ROC_AUC_Python in 720725_GR_ant, 0.856 ± 0.029 for ROC_AUC_DIGITS in 720725_GR_ant, 0.875 ± 0.031 for ROC_AUC_Python in 1347030_TRHR_ago, 0.886 ± 0.028 for ROC_AUC_DIGITS in 1347030_TRHR_ago, 0.879 ± 0.015 for ROC_AUC_Python in 1347032_TGF_beta_ant, 0.907 ± 0.020 for ROC_AUC_DIGITS in 1347032_TGF_beta_ant, 0.762 ± 0.044 for BAC_Python in 720725_GR_ant, 0.791 ± 0.023 for BAC_DIGITS in 720725_GR_ant, 0.811 ± 0.032 for BAC_Python in 1347030_TRHR_ago, 0.829 ± 0.023 for BAC_DIGITS in 1347030_TRHR_ago, 0.805 ± 0.015 for BAC_Python in 1347032_TGF_beta_ant, 0.849 ± 0.030 for BAC_DIGITS in 1347032_TGF_beta_ant, 0.248 ± 0.065 for MCC_Python in 720725_GR_ant, 0.282 ± 0.030 for MCC_DIGITS in 720725_GR_ant, 0.141 ± 0.017 for MCC_Python in 1347030_TRHR_ago, 0.155 ± 0.022 for MCC_DIGITS in 1347030_TRHR_ago, 0.309 ± 0.025 for MCC_Python in 1347032_TGF_beta_ant, 0.384 ± 0.044 for MCC_DIGITS in 1347032_TGF_beta_ant, and 0.790 ± 0.058 for Acc_Python in 720725_GR_ant, 0.812 ± 0.044 for Acc_DIGITS in 720725_GR_ant, 0.781 ± 0.030 for Acc_Python in 1347030_TRHR_ago, 0.769 ± 0.060 for Acc_DIGITS in 1347030_TRHR_ago, 0.770 ± 0.029 for Acc_Python in 1347032_TGF_beta_ant, 0.833 ± 0.033 for Acc in 1347032_TGF_beta_ant, respectively (Table 3).The highest prediction-performance values of ROC_AUC on the valid dataset for the angles and data-split ratios were 0.926 at 185° and train:valid:test = 7:1:2 for Python in 720725_GR_ant, 0.910 at 95° and train:valid:test = 5:5:1 for DIGITS in 720725_GR_ant, 0.915 at 176° and train:valid:test = 3:1:2 for Python in 1347030_TRHR_ago, 0.918 at 185° and train:valid:test = 5:3:4 for DIGITS in 1347030_TRHR_ago, 0.911 at 185° and train:valid:test = 7:1:2 for Python in 1347032_TGF_beta_ant, 0.932 at 75° and train:valid:test = 5:3:2 for DIGITS in 1347032_TGF_beta_ant (Figure 1 and Figure 2; Table 3). Additionally, the highest prediction-performance values of BAC on the valid dataset for the angles and data-split ratios were 0.864 at 185° and train:valid:test = 7:1:2 for Python in 720725_GR_ant, 0.837 at 95° and train:valid:test = 3:3:1 for DIGITS in 720725_GR_ant, 0.868 at 176°and train:valid:test = 3:1:2 for Python in 1347030_TRHR_ago, 0.876 at 355° and train:valid:test = 5:3:4 for DIGITS in 1347030_TRHR_ago, 0.844 at 185° and train:valid:test = 7:1:2 for Python 1347032_TGF_beta_ant, 0.930 at 176° and train:valid:test = 5:3:2 for DIGITS in 1347032_TGF_beta_ant (Figure 3 and Figure 4; Table 3). The highest prediction-performance values of MCC on the valid dataset for the angles and data-split ratios were 0.451 at 176° and train:valid:test = 7:1:2 for Python in 720725_GR_ant, 0.354 at 75° and train:valid:test = 4:4:1 for DIGITS in 720725_GR_ant, 0.473 at 185° and train:valid:test = 7:1:1 for Python in 1259395_TSHR_ant, 0.623 at 75° and train:valid:test = 5:5:1 for DIGITS in 1259395_TSHR_ant, 0.194 at 176° and train:valid:test = 3:1:2 for Python in 1347030_TRHR_ago, 0.876 at 355° and train:valid:test = 5:3:4 for DIGITS in 1347030_TRHR_ago, 0.208 at 355° and train:valid:test = 1:1:1 for Python in 1347032_TGF_beta_ant, 0.478 at 165° and train:valid:test = 2:2:1 for DIGITS in 1347032_TGF_beta_ant (Figures S1 and S2; Table 3). Furthermore, the highest prediction-performance values of Acc on the valid dataset 720725_GR_ant for the angles and data-split ratios were 0.917 at 176° and train:valid:test = 7:1:2 for Python in, 0.939 at 155° and train:valid:test = 4:4:1 for DIGITS in 720725_GR_ant, 0.856 at 176° and train:valid:test = 3:1:2 for Python in 1347030_TRHR_ago, 0.902 at 125° and train:valid:test = 1:1:1 for DIGITS in 1347030_TRH R_ago, 0.834 at 176° and train:valid:test = 7:1:2 for Python in 1347032_TGF_beta_ant, 0.896 at 165° and train:valid:test = 2:2:1 for DIGITS in 1347032_TGF_beta_ant (Figures S3 and S4; Table 3). Addtionally, DeepSnap_Python in the three MIE targets indicated prediction performances of loss, PR_AUC, and F as follows. The mean loss values on the train and valid datasets were 0.413 ± 0.153 for loss_train in 720725_GR_ant and 0.383 ± 0.115 for loss_valid in 720725_GR_ant, 0.247 ± 0.088 for loss_train in 1347030_TRHR_ago and 0.189 ± 0.070 for loss_valid in 1347030_TRHR_ago, 0.280 ± 0.120 for loss_train in 1347032_TGF_beta_ant and 0.316 ± 0.061 for loss_valid in 1347032_TGF_beta_ant (Figures S5–S8; Table 3). The mean PR_AUC values on the valid dataset were 0.335 ± 0.117 in 720725_GR_ant, 0.103 ± 0.041 in 1347030_TRHR_ago, and 0.315 ± 0.056 in 1347032_TGF_beta_ant (Figures S9 and S10; Table 3). The mean F values on the valid dataset were 0.853 ± 0.039 in 720725_GR_ant, 0.868 ± 0.020 in 1347030_TRHR_ago, and 0.833 ± 0.020 in 1347032_TGF_beta_ant (Figures S11 and S12; Table 3). Further, the lowest prediction performance values of loss on the train and valid datasets for the angles and data-split ratios were 0.038 at 176° and train:valid:test = 2:2:1 and 0.110 at 185° and train:valid:test = 7:1:2 in 720725_GR_ant; 0.047 at 176° and train:valid:test = 1:1:1 and 0.194 at 176° and train:valid:test = 3:1:2 in 1347030_TRHR_ago; and 0.044 at 176° and train:valid:test = 1:1:1 and 0.197 at 350° and train:valid:test = 3:2:1 in 1347032_TGF_beta_ant (Figures S5–S8; Table 3).

The highest prediction performance values of PR_AUC on the valid dataset for the angles and data-split ratios were 0.660 at 176° and train:valid:test = 7:1:2 in AID:720725_GR_ant, 0.194 at 176° and train:valid:test = 3:1:2 in 1347030_TRHR_ago, and 0.453 at 176° and train:valid:test = 3:1:1 in 1347032_TGF_beta_ant (Figures S9 and S10; Table 3). In addition, the highest prediction performance values of F on the valid dataset for the angles and data-split ratios were 0.935 at 176° and train:valid:test = 7:1:2 in 720725_GR_ant, 0.914 at 176° and train:valid:test = 3:1:2 in 1347030_TRHR_ago, and 0.876 at 176° and train:valid:test = 7:1:2 in 1347032_TGF_beta_ant (Figures S11 and S12; Table 3). In this study, we observed two performance peaks in prediction models at 176°and 355°of angles in DeepSnap, according to previous results [53,54].

These findings suggested that image augmentation is effectively worked. It has been reported that even though a small number of images was used, the DL can classify by increasing the number of images with the addition of artificial operations, such as movement, rotation, enlargement/reduction, and inversion to the original images [55,56]. In addition, it is known that in conformation generation using algorithms other than MMFF for the force field, the 3D structure differs significantly depending on the algorithm. Therefore, further performance improvement can be expected using other force field calculation algorithms. Further, as a result of examining the depiction condition for ball-and-stick models in the DeepSnap, it was previously reported that the performance can be improved by adjusting the bond thickness and atom color [50].

However, since the image will be similar to the original image, the risk of overfitting, i.e., a decrease in the performance on the test dataset due to the prediction model fitting to match into the training dataset, cannot be ruled [57,58,59,60,61,62,63,64,65,66,67,68]. Thus, data augmentation effectively enables learning with a small number of data. However, suppose it is required to use complex models or obtain high performance to avoid overfitting. In that case, it is important to use high-quality data with few biased features and a sufficiently large data size. There are mainly two methods of data augmentation: offline and online augmentation (also called on-the-fly augmentation), depending on the augmentation timing [69,70]. The offline augmentation is the rotation conversion added to each image in the dataset, doubled in size with the increase in the capacity because the converted image is created for each image. The online augmentation applied to mini-batch that split the dataset into multiple datasets, where the capacity of the dataset does not increase and different random images are generated if the DL is performed using multiple epochs in the same mini-batch. Additionally, this DeepSnap-python has a built-in early stopping function that can the effects of epochs as well as overfit. Therefore, the performance of this system could be more improved by combination of these functions with parameter optimization.

### 2.2. LR and BS in DeepSnap-DL

**Figure 5:**
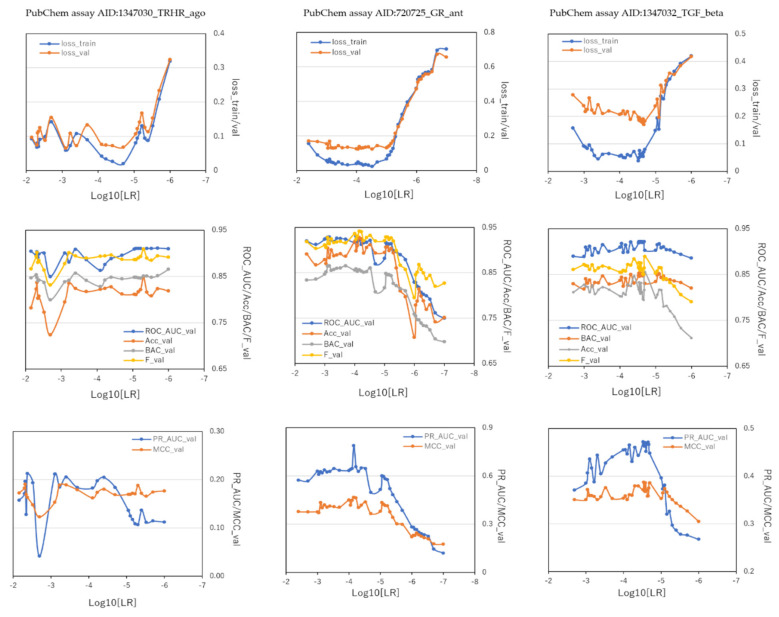
Performance contribution of learning rates (LRs) in DeepSnap−DL using TensorFlow of prediction models of the glucocorticoid receptor (PubChem assay AID:720725_GR_ant), TGF-beta/Smad (PubChem assay AID:1347032_TGF_beta_ant), and agonist of the thyrotropin-releasing hormone receptor (PubChem assay AID:1347030_TRHR_ago) in the validation dataset; *n* = 2.

To investigate the effect of hyperparameters in DeepSnap-DL with Python system on prediction-performance values of the three MIE targets, we optimized 39 LRs from 0.004 to 0.0000001 in 720725_GR_ant, 24 LRs from 0.007 to 0.000001 in 1347030_TRHR_ago, and 38 LRs from 0.002 to 0.000001 in 1347032_TGF_beta_ant using the valid dataset (Table S2). The mean ROC_AUC, BAC, MCC, and Acc values in the valid dataset were 0.884 ± 0.930 for ROC_AUC in 720725_GR_ant, 0.897 ± 0.016 for ROC_AUC in 1347030_TRHR_ago, 0.909 ± 0.011 for ROC_AUC in 1347032_TGF_beta_ant, 0.817 ± 0.053 for BAC in 720725_GR_ant, 0.844 ± 0.012 for BAC in 1347030_TRHR_ago, 0.839 ± 0.010 for BAC in 1347032_TGF_beta_ant, 0.354 ± 0.090 for MCC in 720725_GR_ant, 0.171 ± 0.015 for MCC in 1347030_TRHR_ago, 0.361 ± 0.016 for MCC in 1347032_TGF_beta_ant, and 0.859 ± 0.060 for Acc in 720725_GR_ant, 0.881 ± 0.025 for Acc in 1347030_TRHR_ago, 0.807 ± 0.028 for Acc in 1347032_TGF_beta_ant, respectively (Table 4). The highest prediction-performance values of ROC_AUC on the valid dataset for LRs were 0.930 at 0.00009 in 720725_GR_ant, 0.911 at 0.000002 in 1347030_TRHR_ago, 0.922 at 0.000021 in 1347032_TGF_beta_ant (Figure 5, Table 4). Additionally, the highest prediction-performance values of BAC on the valid dataset for LRs were 0.865 at 0.0007 in 720725_GR_ant, 0.865 at 0.000001 in 1347030_TRHR_ago, 0.853 at 0.000029 in 1347032_TGF_beta_ant (Figure 5, Table 4). The highest prediction-performance values of MCC on the valid dataset for LRs were 0.466 at 0.00007 in 720725_GR_ant, 0.191 at 0.0048 in 1347030_TRHR_ago, 0.387 at 0.000029 in 1347032_TGF_beta_ant (Figure 5, Table 4). Furthermore, the highest prediction-performance values of Acc on the valid dataset for LRs were 0.928 at 0.00007 in 720725_GR_ant, 0.848 at 0.000005 in 1347030_TRHR_ago, 0.855 at 0.00002 in 1347032_TGF_beta_ant (Figure 5, Table 4).

DeepSnap_Python in the three MIE targets achieved the following prediction-performance values of loss, PR_AUC, and F. The mean loss values on the train and valid datasets were 0.215 ± 0.231 for loss_train in 720725_GR_ant and 0.263 ± 0.186 for loss_valid in 720725_GR_ant, 0.098 ± 0.062 for loss_train in AID: 1347030_TRHR_ago and 0.122 ± 0.058 for loss_valid in 1347030_TRHR_ago, 0.125 ± 0.110 for loss_train in 1347032_TGF_beta_ant and 0.236 ± 0.062 for loss_valid in 1347032_TGF_beta_ant (Figure 5, Table 4). Additionally, the mean PR_AUC values on the valid dataset were 0.502 ± 0.177 in 720725_GR_ant, 0.155 ± 0.045 in 1347030_TRHR_ago, 0.410 ± 0.064 in 1347032_TGF_beta_ant (Figure 5, Table 4). The mean F values on the valid dataset were 0.898 ± 0.039 (PubChem assay AID:720725_GR_ant), 0.886 ± 0.015 in 1347030_TRHR_ago, 0.858 ± 0.019 in 1347032_TGF_beta_ant (Figure 5, Table 4).

Furthermore, the lowest prediction-performance values of loss on the train and valid datasets for the LRs were 0.022 at 0.00003 and 0.124 at 0.00003 in 720725_GR_ant, 0.020 at 0.00002 and 0.066 at 0.0008 in 1347030_TRHR_ago, 0.038 at 0.00003 and 0.170 at 0.000021 in 1347032_TGF_beta_ant (Figure 5, Table 4). The highest prediction-performance values of PR_AUC on the valid dataset for LRs were 0.789 at 0.00007 in 720725_GR_ant, 0.213 at 0.0042 in 1347030_TRHR_ago, 0.472 at 0.00003 in 1347032_TGF_beta_ant (Figure 5, Table 4). Additionally, the highest prediction-performance values of F on the valid dataset for LRs were 0.942 at 0.00007 in 720725_GR_ant, 0.909 at 0.000005, 0.890 at 0.00002 in 1347032_TGF_beta_ant (Figure 5, Table 4).

Finally, to investigate the effect of BS in the improved DeepSnap-DL with Python system on prediction-performance values, we optimized 84 BSs from 2 to 300 in 720725_GR_ant, 13 LRs from 2 to 26 in 1347030_TRHR_ago, and 37 LRs from 2 to 80 in 1347032_TGF_beta_ant using the valid dataset (Table S3). The mean ROC_AUC, BAC, MCC, and Acc values in the test dataset were 0.983 ± 0.032 for ROC_AUC in 720725_GR_ant, 0.929 ± 0.003 for ROC_AUC in 1347030_TRHR_ago, 0.918 ± 0.005 for ROC_AUC in 1347032_TGF_beta_ant, 0.866 ± 0.033 for BAC in 720725_GR_ant, 0.877 ± 0.004 for BAC in 1347030_TRHR_ago, 0.848 ± 0.007 for BAC in 1347032_TGF_beta_ant, 0.444 ± 0.056 for MCC in 720725_GR_ant, 0.194 ± 0.004 for MCC in 1347030_TRHR_ago, 0.368 ± 0.011 for MCC in 1347032_TGF_beta_ant, and 0.908 ± 0.021 for Acc in 720725_GR_ant, 0.855 ± 0.005 for Acc in 1347030_TRHR_ago, 0.810 ± 0.011 for Acc in 1347032_TGF_beta_ant, respectively (Table 5).

The highest prediction-performance values of ROC_AUC on the test dataset for BS were 0.983 at 125 in 720725_GR_ant, 0.934 at 14 in 1347030_TRHR_ago, 0.925 at 28 in 1347032_TGF_beta_ant (Figure S13, Table 5). Additionally, the highest prediction-performance values of BAC on the test dataset for BSs were 0.930 at 125 in 720725_GR_ant, 0.881 at 22 in 1347030_TRHR_ago, 0.862 at 44 in 1347032_TGF_beta_ant (Figure S13, Table 5). The highest prediction-performance values of MCC on the test dataset for BSs were 0.604 at 200 in 720725_GR_ant, 0.200 at 14 in 1347030_TRHR_ago, 0.390 at 28 in 1347032_TGF_beta_ant (Figure S13, Table 5). Furthermore, the highest prediction-performance values of Acc on the test dataset for BSs were 0.954 at 200 in 720725_GR_ant, 0.863 at 14 in 1347030_TRHR_ago, 0.835 at 20 in 1347032_TGF_beta_ant (Figure S13, Table 5).

Additionally, DeepSnap_Python in the three MIE targets achieved the following prediction-performance values of loss, PR_AUC, and F. The mean loss values on the train and test datasets were 0.045 ± 0.033 for loss_train in 720725_GR_ant and 0.119 ± 0.025 for loss_test in 720725_GR_ant, 0.322 ± 0.013 for loss_train in 1347030_TRHR_ago and 0.314 ± 0.022 for loss_test in 1347030_TRHR_ago, 0.097 ± 0.047 for loss_train in 1347032_TGF_beta_ant and 0.203 ± 0.023 for loss_test in 1347032_TGF_beta_ant (Figure S13, Table 5). Additionally, the mean PR_AUC values on the test dataset were 0.654 ± 0.087 in 720725_GR_ant, 0.136 ± 0.011 in 1347030_TRHR_ago, 0.431 ± 0.032 in 1347032_TGF_beta_ant (Figure S13, Table 5). The mean F values on the test dataset were 0.930 ± 0.014 in 720725_GR_ant, 0.914 ± 0.003 in 1347030_TRHR_ago, 0.860 ± 0.008 in 1347032_TGF_beta_ant (Figure S13, Table 5). Furthermore, the lowest prediction-performance values of loss on the train and test datasets for BSs were 0.019 at 48 and 0.073 at 120 in 720725_GR_ant, 0.301 at 14 and 0.255 at 2 in 1347030_TRHR_ago, 0.037 at 20 and 0.172 at 34 in 1347032_TGF_beta_ant (Figure S13, Table 5). The highest prediction-performance values of PR_AUC on the test dataset for BSs were 0.800 at 290 in 720725_GR_ant, 0.154 at 14 in 1347030_TRHR_ago, 0.476 at 28 in 1347032_TGF_beta_ant (Figure S13, Table 5). Additionally, the highest prediction-performance values of F on the test dataset for BSs were 0.961 at 200 in 720725_GR_ant, 0.919 at 14, 0.877 at 20 in 1347032_TGF_beta_ant (Figure S13, Table 5).

As a method often used to improve the generalization performance of DL, LR decay, meaning to lower LR in places where learning has progressed to some extent, is known to improve accuracy sharply [71]. However, their behavior changes significantly depending on datasets, network types, and optimization methods. Therefore, a function that automatically attenuates the LR decay is required when learning converges to some extent. Thus, the improved DeepSnap-DL method was added with an early stopping function to extract models with the highest performance in a series of learning processes by discontinuing learning before entering the overfitting phase; thereby shortening the learning time.

It was previously reported that BS and LR are proportional, whereas BS and momentum coefficient are inversely proportional [72]. It is considered that the learning converges to the sharp minimum as BS increases. Meanwhile, when BS is small, larger variance positively affects the performance in DL, such as regularization. However, it was shown that there was an optimal BS for LRs, suggesting that it is essential to have an appropriate BS within that LRs, instead of reducing the BS. Thus, considering the learning efficiency, it is appropriate to set the BS sufficiently large and adjust the LR.

These findings are expected to lead to drug development from the estimation and identification of new ligands for nuclear receptors.

## 3. Materials and Methods

### 3.1. Data

The datasets of three MIE targets, including antagonists of the glucocorticoid receptor (PubChem assay AID:720725_GR_ant), TGF-beta/Smad (PubChem assay AID:1347032_TGF_beta_ant), and agonist of the thyrotropin-releasing hormone receptor (PubChem assay AID:1347030_TRHR_ago) for the chemical structures in SMILES format and the corresponding agonist or antagonist scores defined as Pubchem_activity_scores from the Tox21 10K library in the PubChem database housing quantitative high-throughput assays to identify small molecule agonists and antagonists for MIEs, as previously reported, were downloaded [50,51,52,53,54] (Table S1a–c). After eliminating overlapping chemicals and inorganic compounds because of the presence of possible stereoisomers or salts, we defined active and inactive compounds by activity scores, which the agonist and antagonist scores ranged from 0% to 100% by normalizing each titration point relative to the positive control compound and dimethyl sulfoxide (DMSO)-only wells according to the following equation: % activity = [(Vcompound − Vdmso)/(Vpos − Vdmso)] × 100, where Vcompound, Vdmso, and Vpos denote the compound-well values, median values of the DMSO-only wells, and median values of the positive control well in the reporter gene assay, i.e., active and inactive compounds were defined by activity scores 40–100 and 0–39, respectively (Table S1a–c). The mean number of chemicals was 7601 ± 63, and the highest and lowest numbers of chemicals were 7662 in 1347030_TRHR_ago and 7539 in 720725_GR_ant, respectively (Table S1a–c and Table 1). Further, we divided the data for the chemical compounds into two groups based on their activity scores: active and inactive chemicals. Active chemicals had an activity score ≥ 40, whereas inactive chemicals had an activity score < 40. The mean numbers and percentages of active chemicals among three MIEs were 248 ± 167 and 3.27 ± 2.20, and the highest and lowest numbers and percentages of active chemicals were, respectively, 395 and 5.19% for 1347032_TGF_beta and 67 and 0.87% for 1347030_TRHR_ago (Table 6). Data were divided into train, valid, and test datasets. The first two datasets were used for training and fine-tuning the prediction models. The final evaluation of the constructed models was performed using a foldout test dataset.

### 3.2. DeepSnap

We applied the SMILES format for 3D conformational import to generate the 3D chemical database with rotatable torsion and saved it as a structure data file (SDF) using molecular operating environment (MOE) 2018 scientific applications (MOLSIS Inc., Tokyo, Japan). Then, the external program, CORINA classic software (Molecular Network GmbH, Nürnberg, Germany, https://www.mn-am.com/products/corina, accessed on 25 January 2022) was used to determine a suitable form of each chemical structure. The 3D chemical structures of the compounds from SDF files were depicted as 3D ball-and-stick models with different colors corresponding to different atoms by a Jmol, an open-source Java viewer software for 3D molecular modeling of chemical structures [73,74,75]. The 3D-chemical models were automatically captured as snapshots of user-defined angle increments on the *x*-, *y*-, and *z*-axes saved as 256 × 256-pixel resolution PNG files (RGB) and split into three train, valid, and test datasets, as previously reported [50,51,52,53,54]. All PNG image files produced by DeepSnap were resized using NVIDIA DL GPU training system (DIGITS) version 4.0.0 software (NVIDIA, Santa Clara, CA, USA), on four-GPU systems, Tesla-V100-PCIE (31.7 GB) with 256 × 256-pixel resolution as input data, as previously reported [50,51,52]. We used a pre-trained open-source DL model, Caffe, that the network of GoogLeNet consisted of deep convolutional neural network (CNN) architectures comprised complex inspired by LeNet, on the CentOS Linux distribution 7.3.1611. At the DeepSnap-DL-DIGITS method, the prediction models were constructed by train datasets using 30 epochs in DL. Among these epochs, the lowest loss value in the valid dataset was selected for the next examination for prediction using the test dataset.

The improved DeepSnap-DL-Python system used a new 3D conformational import application, called SMILES_TO_SDF, to produce the SDF files from the SMILES format. We used PyMOL, an open-source molecular visualization system written in the Python programming language (Schrödinger, Inc., New York, NY, USA), to obtain high-quality 3D molecular modeling of chemical structures with 3D ball-and-stick models with different colors corresponding to different atoms. The 3D chemical structures can produce different images depending on the direction. They are captured automatically by DeepSnap as snapshots with user-defined angle increments with respect to the *x*-, *y*-, and *z*-axes as the DeepSnap-DL-DIGITS method. The snapshots, saved as 256 × 256-pixel PNG files (RGB), were divided into the train, valid, and test datasets. Additionally, the external test dataset is permanently fixed. TensorFlow and Keras on CentOS Linux 7.3.1611 with the CNN GoogLeNet were used all 2D PNG images produced by the DeepSnap-DL-Python system for training and fine-tuning the prediction models. Background colors in the images were changed to the color values in PyMOL, where a force field, which is a set of parameters for the bond lengths, angles, torsional parameters, electrostatic properties, and van der Waals interactions, uses the Merck Molecular Force Field (MMFF) [76].

Next, using the structural information for these chemicals derived from the SMILES format, the 3D chemical structure per compound with “rotatable torsions” was depicted using MOE application software program, and optimized to generate a single low energy conformation using CORINA classic software. These 3D chemical structures were saved in SDF format as a database file. Then, molecular images were generated as snapshots of the 3D structure from the SDF file using the DeepSnap method at different angles along the *x*, *y*, and *z* axes. The prediction models of the three MIE targets were constructed using these images of the 3D chemicals as input data for the DIGITS-based DL. Another system that is modified DeepSnap-DL by TensorFlow and Keras with Python was used. The SMILES format was used for a new 3D application, called SMILES_TO_SDF, to produce high-quality 3D molecular modeling of the chemical structures saved as a chemical database in SDF format. 2D PNG images produced from the SDF file were produced by DeepSnap, and the prediction models were constructed using these images as input data by DL with TensorFlow and Keras, called DeepSnap-DL-Python.

### 3.3. Evaluation of Prediction Models

We analyzed the probability of the prediction results using the prediction model with the lowest minimum loss in valid value among 30 examined echoes using the DeepSnap-DL-DIGITS method. We used the medians of each predicted value as representative values for target molecules using statistical analysis software JMP^®^ Pro. 14 (SAS Institute Inc., Cary, NC, USA), as previously reported [50,51,52], because the process of the DeepSnap-DL-DIGITS method calculated the probabilities for each image prepared from different angles with the *x*-, *y*-, and *z*-axes directions for one molecule. Classification performance was evaluated based on a confusion matrix defined by the cutoff value (θ) from the Youden’s Index (YI) as follows [77,78,79]:

$$YI=\max\overset{k-1}{\underset{j=1}{\sum }}wjFj\left(\theta \right)-\left(1-wj\right)Fj+1\left(\theta j\right)$$

$$\theta =\arg\max\overset{k-1}{\underset{j=1}{\sum }}\left(1-wj\right)Fj+1\left(\theta j\right)-\mathrm{wj}Fj\left(\theta j\right)$$

where *k* is the diagnostic categories, wj ∈ (0,1).

However, the DeepSnap-DL-Python system automatically obtains the probability of prediction results with the lowest minimum loss_valid value among 30 examined epochs, which are the numbers of repeats for one training dataset modulated by early stopping. Additionally, the performance of each model was automatically calculated in terms of the metrics: ROC_AUC, precision recall_AUC (PR_AUC), balanced accuracy (BAC), F, Matthew’s correlation coefficient (MCC), accuracy (Acc), and loss. These performance metrics are defined as follows. Here TP, FN, TN, and FP denote true positive, false negative, true negative, and false positive, respectively.

Sensitivity = ΣTPs/(ΣTPs + ΣFNs)

Specificity = ΣTNs/(ΣTNs + ΣFPs)

BAC = (sensitivity + specificity)/2

Acc = Accuracy = (TP + TN)/(TP + FP + TN + FN)

Precision = TP/(TP + FP)

Recall = TP/(TP + FN)

F-measure (F) = 2 × Recall × Precision/(Recall + Precision)

$$\mathrm{MCC}=(\mathrm{TP}\times \mathrm{TN}-\mathrm{FP}\times \mathrm{FN})/\sqrt{\left(\mathrm{TP}+\mathrm{FP}\right)\times \left(\mathrm{TP}+\mathrm{FN}\right)\times \left(\mathrm{TN}+\mathrm{FP}\right)\times \left(\mathrm{TN}+\mathrm{FN}\right)}.$$

To determine the optimal cutoff point for the definition of TP, FN, TN, and FP, we adopted a method for maximizing the sensitivity (1—specificity), called YI. This index has a value ranging from 0 to 1, where 1 represents the maximum effectiveness, and 0 represents the minimum effectiveness. Additionally, the area under the curve (AUC) for the receiver operating characteristics (ROC) is given by

$$\mathrm{ROC}\text{\_}\mathrm{AUC}=1/Np\sum_{j=1}^{Np}f\left(j\right)$$

$$fj=1/T\sum_{t=1}^{T}Wt\left\{\begin{array}{c} 1\;if\;pj≧t\\ 0\;otherwise\; \end{array}\right\}$$

*Wt* = 1/2 (prec*_t +_*_1_ − prec*_t −_*_1_)

$${\mathrm{prec}}_{t}=\left(\#\;\mathrm{of}\;\mathrm{points}\;i\;\mathrm{where}\;\mathrm{pi}\;≧t\;and\;ci=1\right)/\left(\#\;\mathrm{of}\;\mathrm{points}\;i\;\mathrm{where}\;\mathrm{pi}\;≧t\right)\;$$

Here, ROC_AUC denotes AUC *f*, *j* iterates over the true points, *Np* is the number of true points, *T* is the number of thresholds, and prec*t* is the precision at threshold *t*. For broader cases, let prec0 = prec1, and precT = 0 [80]. The PR curve is the plot of Recall (x) vs. Precision (y), and PR_AUC was calculated according to previous studies [53,54]. This study used N = 3 to reduce the bias, and the values are represented as averages.

## 4. Conclusions

In this study, we constructed prediction models for antagonists of the glucocorticoid receptor, TGF-beta/Smad, and agonist of the thyrotropin-releasing hormone receptor using the classic DeepSnap-DL system with DIGITS and improved DeepSnap-DL system with TensorFlow and Keras using the Tox21 10k library. We performed high-throughput and decreased computational costs using the improved DeepSnap-DL system by optimizing the parameters in DeepSnap. Consequently, we obtained that the improved DeepSnap-DL system would be a powerful advanced QSAR system on toxicological and biochemical/cheminformatic fields.

## Figures and Tables

**Table 1:** Angles used in DeepSnap on modeling of the three MIE targets.

	Angles in DeepSnap_Python			Angles in DeepSnap_DIGITS		
PubChem Assay AID	No.	Minimum (°)	Maximum (°)	No.	Minimum (°)	Maximum (°)
720725_GR_ant	31	65	350	23	70	345
1347030_TRHR_ago	15	95	325	17	95	355
1347032_TGF_beta	16	75	350	16	75	350

**Table 2:** Data splits used in DeepSnap on modeling of the three MIE targets.

		Data Splits in DeepSnap_Python		Data Splits in DeepSnap_DIGITS
PubChem Assay AID	No.	Type	No.	Type
720725_GR_ant	7	1:1:1, 2:2:1, 3:3:1, 4:4:1, 5:5:1, 5:3:2, 7:1:2	7	1:1:1, 2:2:1, 3:3:1, 4:4:1, 5:5:1, 5:3:2, 7:1:2
1347030_TRHR_ago	3	1:1:1, 3:1:2, 5:3:4	3	1:1:1, 3:1:2, 5:3:4
1347032_TGF_beta	8	1:1:1, 2:2:1, 3:1:1, 3:2:1, 5:3:2, 5:5:1, 6:1:2, 7:1:2	8	1:1:1, 2:2:1, 3:1:1, 3:2:1, 5:1:1, 5:3:2, 6:1:2, 7:1:2

**Table 3:** Performances with angles using two DeepSnap systems of the four MIE targets.

PubChem		720725_GR_Ant		1347030_TRHR_Ago		1347032_TGF_Beta_Python	
Assay AID		Python	DIGITS	Python	DIGITS	Python	DIGITS
ROC_AUC	average	0.832 ± 0.048	0.856 ± 0.029	0.875 ± 0.031	0.886 ± 0.028	0.879 ± 0.015	0.907 ± 0.020
	max_ROC_AUC	0.926	0.910	0.915	0.918	0.911	0.932
	max_angle	185	95	176	185	185	75
	max_split	7:1:2	5:5:1	3:1:2	5:3:4	7:1:2	5:3:2
BAC	average	0.762 ± 0.044	0.791 ± 0.023	0.811 ± 0.032	0.829 ± 0.023	0.805 ± 0.015	0.849 ± 0.030
	max_BAC	0.864	0.837	0.868	0.876	0.844	0.930
	max_angle	185	95	176	355	185	176
	max_split	7:1:2	3:3:1	3:1:2	5:3:4	7:1:2	5:3:2
MCC	average	0.248 ± 0.065	0.282 ± 0.030	0.141 ± 0.017	0.155 ± 0.022	0.309 ± 0.025	0.384 ± 0.044
	max_MCC	0.451	0.354	0.194	0.208	0.373	0.478
	max_angle	176	75	176	355	176	165
	max_split	7:1:2	4:4:1	3:1:2	1:1:1	7:1:2	2:2:1
Acc	average	0.790 ± 0.058	0.812 ± 0.044	0.781 ± 0.030	0.769 ± 0.060	0.770 ± 0.029	0.833 ± 0.033
	max_Acc	0.917	0.939	0.856	0.902	0.834	0.896
	max_angle	176	155	176	125	176	165
	max_split	7:1:2	4:4:1	3:1:2	1:1:1	7:1:2	2:2:1
loss_val	average	0.383 ± 0.115	0.108 ± 0.014	0.189 ± 0.070	0.032 ± 0.007	0.316 ± 0.061	0.113 ± 0.011
	min_loss_train	0.110	0.065	0.194	0.024	0.197	0.087
	min_angle	185	195	176	325	350	230
	max_split	7:1:2	7:1:2	3:1:2	3:1:2	3:2:1	7:1:2
loss_train	average	0.413 ± 0.153		0.247 ± 0.088		0.280 ± 0.120	
	min_loss_train	0.038		0.047		0.044	
	min_angle	176		176		176	
	max_split	2:2:1		1:1:1		1:1:1	
PR_AUC	average	0.335 ± 0.117		0.103 ± 0.041		0.315 ± 0.056	
	max_PR_AUC	0.660		0.194		0.453	
	max_angle	176		176		176	
	max_split	7:1:2		3:1:2		3:1:1	
F	average	0.853 ± 0.039		0.868 ± 0.020		0.833 ± 0.020	
	max_F	0.935		0.914		0.876	
	max_angle	176		176		176	
	max_split	7:1:2		3:1:2		7:1:2	

**Table 4:** Performances with LRs using DeepSnap_python system of the three MIE targets.

PubChem		720725_GR_Ant	1347030_TRHR	1347032_TGF_Beta
Assay AID		Train:Valid:Test = 7:1:2	Train:Valid:Test = 3:1:2	Train:Valid:Test = 7:1:2
ROC_AUC	average	0.884 ± 0.053	0.897 ± 0.016	0.909 ± 0.011
	max_ROC_AUC	0.930	0.911	0.922
	max_LR	0.00009	0.000002	0.000021
BAC	average	0.817 ± 0.053	0.844 ± 0.012	0.839 ± 0.010
	max_BAC	0.865	0.865	0.853
	max_LR	0.0007	0.000001	0.000029
MCC	average	0.354 ± 0.090	0.171 ± 0.015	0.361 ± 0.016
	max_MCC	0.466	0.191	0.387
	max_LR	0.00007	0.0048	0.000029
Acc	average	0.859 ± 0.060	0.811 ± 0.025	0.807 ± 0.028
	max_Acc	0.928	0.848	0.855
	max_LR	0.00007	0.000005	0.00002
loss_train	average	0.215 ± 0.231	0.098 ± 0.062	0.125 ± 0.110
	min_loss	0.022	0.020	0.038
	min_LR	0.00003	0.00002	0.00003
loss_val	average	0.263 ± 0.186	0.122 ± 0.058	0.236 ± 0.062
	min_loss	0.124	0.066	0.170
	min_LR	0.00003	0.0008	0.000021
PR_AUC	average	0.502 ± 0.177	0.155 ± 0.045	0.410 ± 0.064
	max_PR_AUC	0.789	0.213	0.472
	max_LR	0.00007	0.0042	0.00003
F	average	0.898 ± 0.039	0.886 ± 0.015	0.858 ± 0.019
	max_F	0.942	0.909	0.890
	max_LR	0.00007	0.000005	0.00002

**Table 5:** Performances with LRs using DeepSnap_python system of the three MIE targets.

PubChem		720725_GR_Ant	1347030_TRHR	1347032_TGF_Beta
Assay AID		Train:Valid:Test = 7:1:2	Train:Valid:Test = 3:1:2	Train:Valid:Test = 7:1:2
ROC_AUC	average	0.983 ± 0.032	0.929 ± 0.003	0.918 ± 0.005
	max_ROC_AUC	0.983	0.934	0.925
	max_BS	125	14	28
BAC	average	0.866 ± 0.033	0.877 ± 0.004	0.848 ± 0.007
	max_BAC	0.930	0.881	0.862
	max_BS	125	22	44
MCC	average	0.444 ± 0.056	0.194 ± 0.004	0.368 ± 0.011
	max_MCC	0.604	0.200	0.390
	max_BS	200	14	28
Acc	average	0.908 ± 0.021	0.855 ± 0.005	0.810 ± 0.011
	max_Acc	0.954	0.863	0.835
	max_BS	200	14	20
loss_train	average	0.045 ± 0.033	0.322 ± 0.013	0.097 ± 0.047
	min_loss	0.019	0.301	0.037
	min_BS	48	14	20
loss_test	average	0.119 ± 0.025	0.314 ± 0.022	0.203 ± 0.023
	min_loss	0.073	0.255	0.172
	min_BS	120	2	34
PR_AUC	average	0.654 ± 0.087	0.136 ± 0.011	0.431 ± 0.032
	max_PR_AUC	0.800	0.154	0.476
	max_BS	290	14	28
F	average	0.930 ± 0.014	0.914 ± 0.003	0.860 ± 0.008
	max_F	0.961	0.919	0.877
	max_BS	200	14	20

**Table 6:** Active and inactive chemical compounds of the three MIE targets.

	All	Active Compound		Inactive Compound	
PubChem Assay AID	No.	No.	%	No.	%
720725_GR_ant	7537	283	3.75	7254	96.25
1347030_TRHR_ago	7662	67	0.87	7595	99.13
1347032_TGF_beta	7604	395	5.19	7209	94.81

## Data Availability

All samples of the SMILES compounds and technicalism are available from the authors.

## Supplementary Materials

The following supporting information can be downloaded at: https://www.mdpi.com/article/10.3390/ijms23042141/s1.
