# Supplementary material for: A Deep Learning-Based Quantitative Structure–Activity Relationship System Construct Prediction Model of Agonist and Antagonist with High Performance
Source: Int J Mol Sci. 2022 Feb 15;23(4):2141. doi: 10.3390/ijms23042141 (PMC8877122; doi:10.3390/ijms23042141)
Supplement: Supplementary file 1 [file ijms-23-02141-s001.zip › ijms-1592658-supplementary Figure.pdf]

International Journal of
Molecular Sciences

MDPI

Figure S1

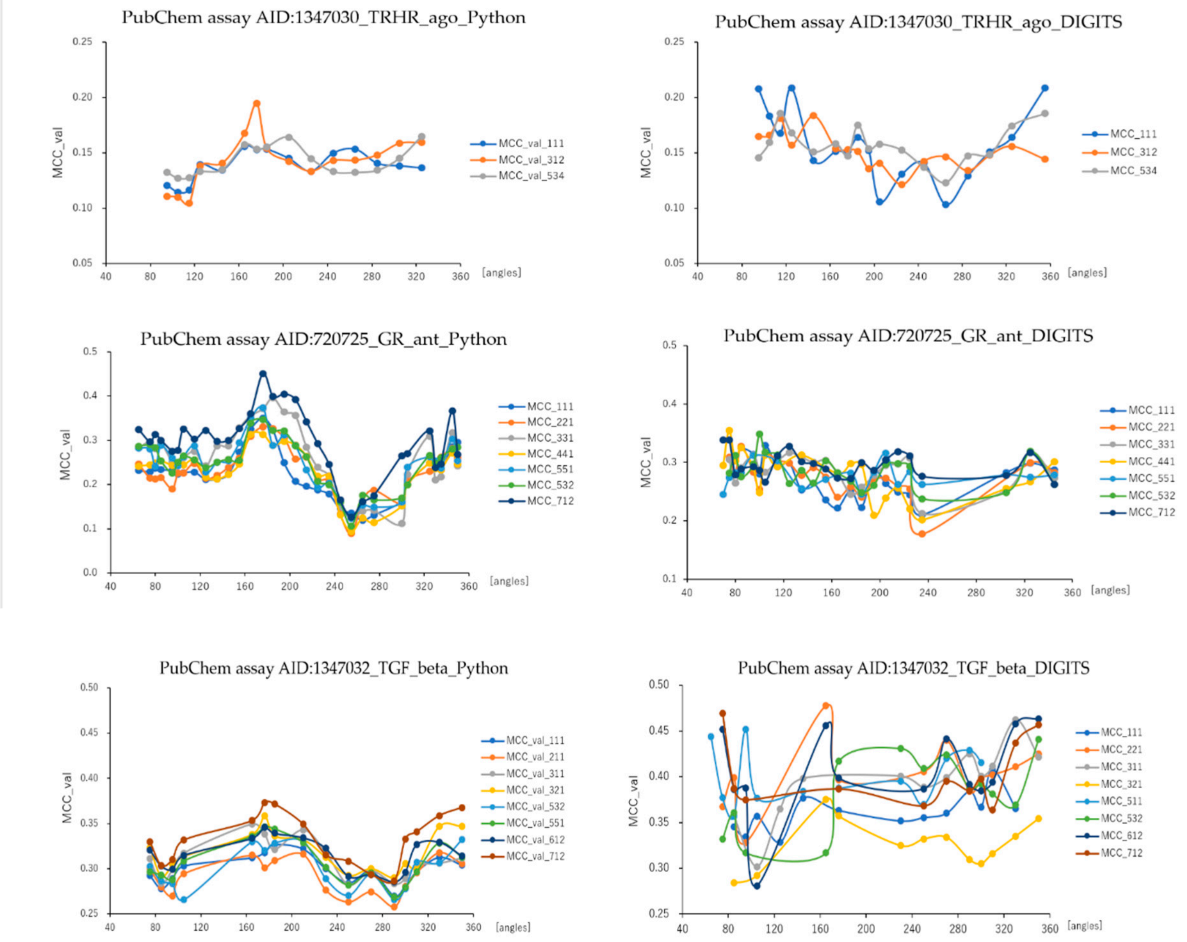

Figure S1. MCC with angles in DeepSnap-DL using TensorFlow and DIGITS of prediction models of the glucocorticoid receptor (PubChem assay AID:720725_GR_ant), TGF-beta/Smad (PubChem assay AID:1347032_TGF_beta_ant), and agonist of the thyrotropin-releasing hormone receptor (PubChem assay AID:1347030_TRHR_ago) in the validation dataset; n = 3.

Figure S2

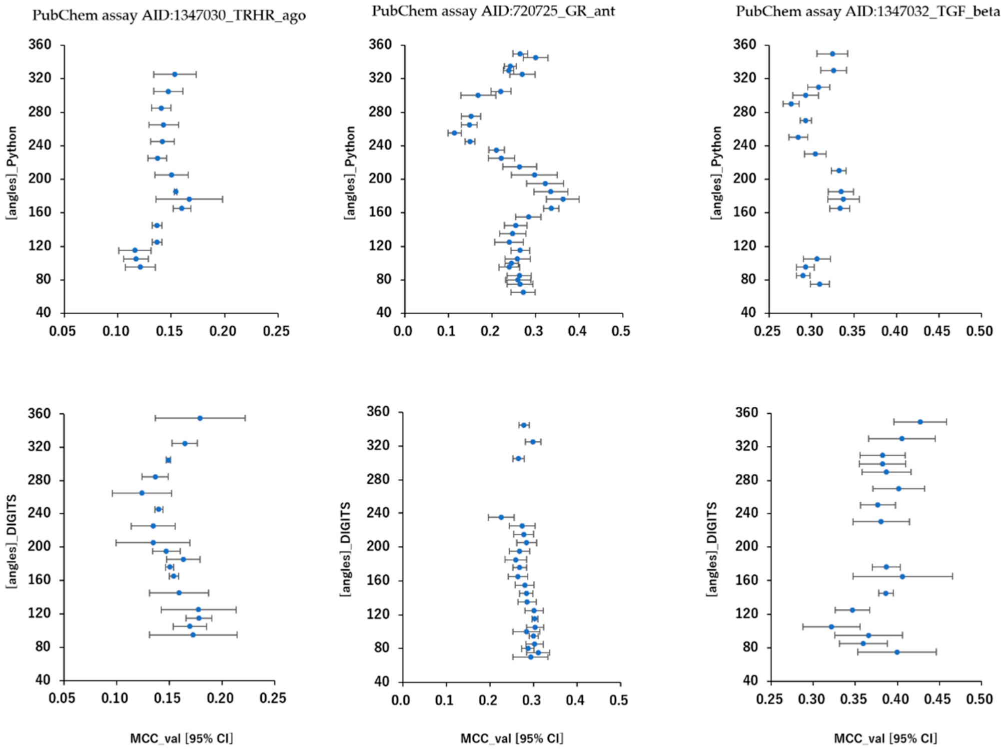

Figure S2. Differences in mean MCC levels shown as dots with 95% confident interval (95% CI) as error bars with angles in DeepSnap-DL using TensorFlow and DIGITS of prediction models of the glucocorticoid receptor (PubChem assay AID:720725_GR_ant), TGF-beta/Smad (PubChem assay AID:1347032_TGF_beta_ant), and agonist of the thyrotropin-releasing hormone receptor (PubChem assay AID:1347030_TRHR_ago) in the validation dataset; n = 3.

Figure S3

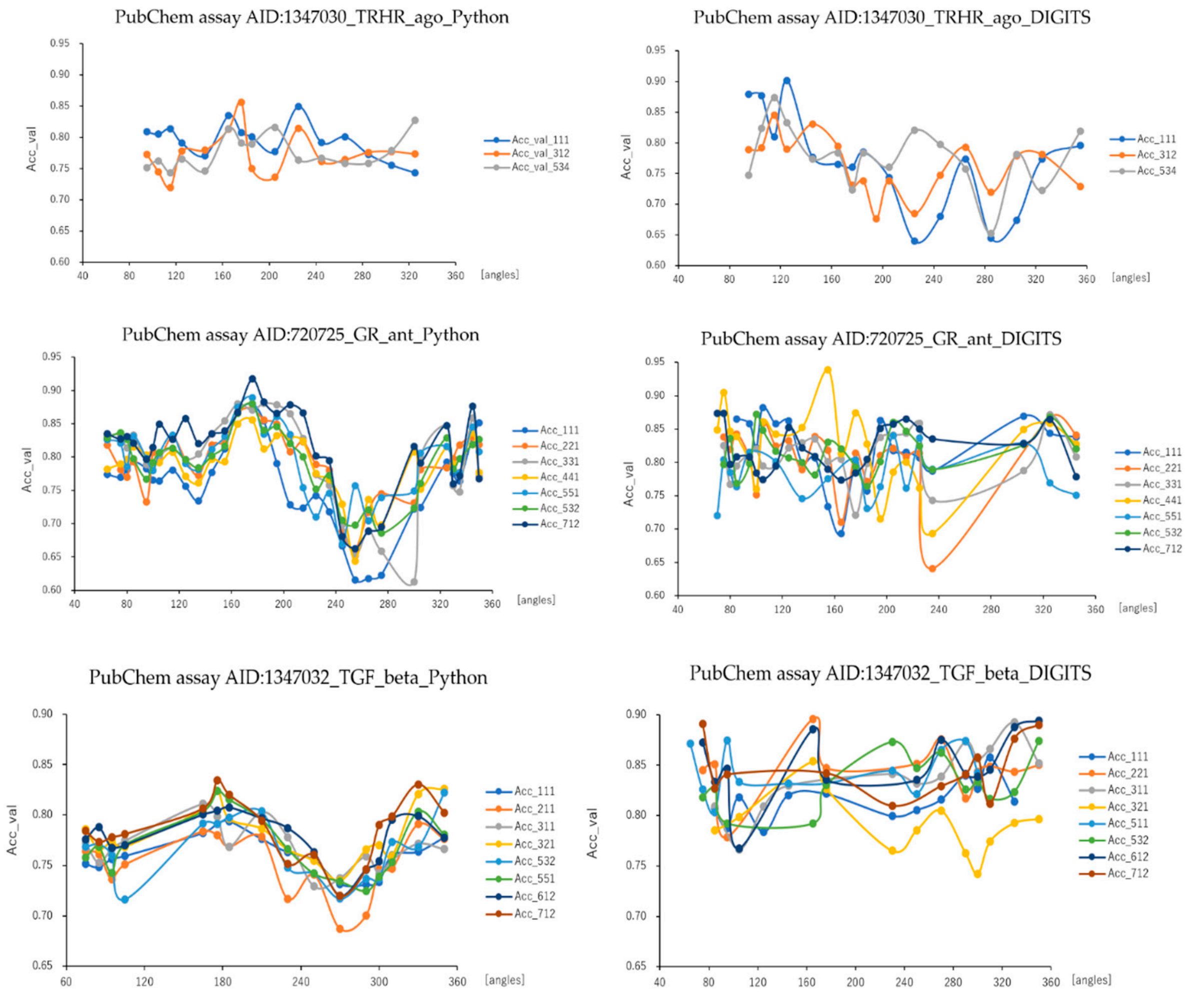

Figure S3. Acc with angles in DeepSnap-DL using TensorFlow and DIGITS of prediction models of the glucocorticoid receptor (PubChem assay AID:720725_GR_ant), TGF-beta/Smad (PubChem assay AID:1347032_TGF_beta_ant), and agonist of the thyrotropin-releasing hormone receptor (PubChem assay AID:1347030_TRHR_ago) in the validation dataset; n=3.

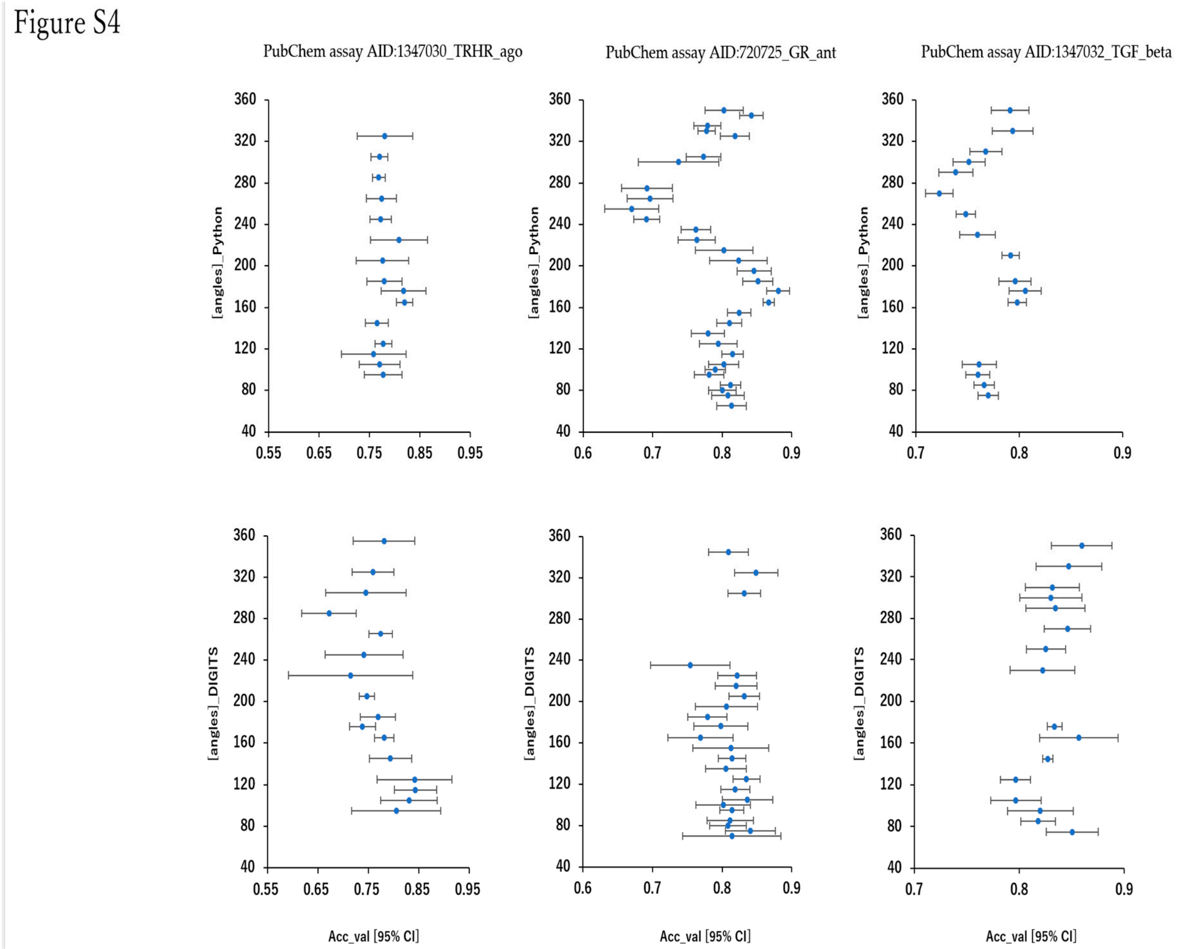

Figure S4. Differences in mean Acc levels shown as dots with 95% confident interval (95% CI) as error bars with angles in DeepSnap-DL using TensorFlow and DIGITS of prediction models of the glucocorticoid receptor (PubChem assay AID:720725_GR_ant), TGF-beta/Smad (PubChem assay AID:1347032_TGF_beta_ant), and agonist of the thyrotropin-releasing hormone receptor (PubChem assay AID:1347030_TRHR_ago) in the validation dataset; n = 3.

Figure S5

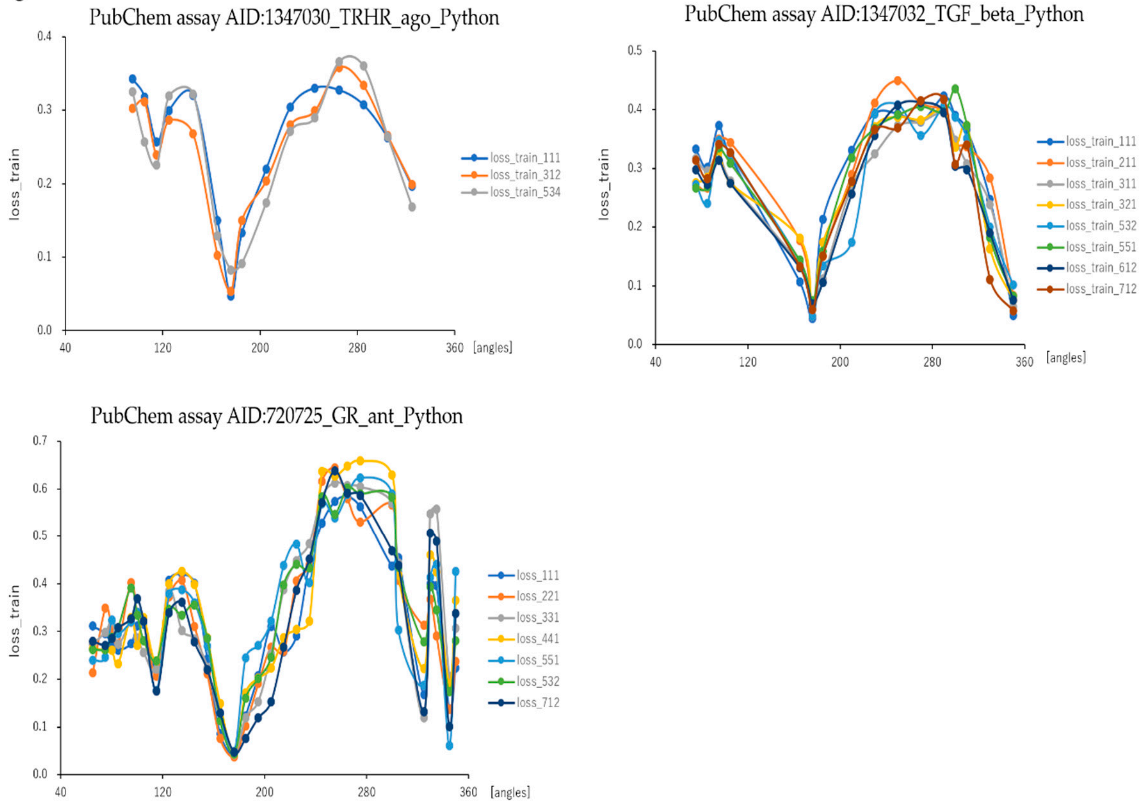

Figure S5. Loss with angles in DeepSnap-DL using TensorFlow and DIGITS of prediction models of the glucocorticoid receptor (PubChem assay AID:720725_GR_ant), TGF-beta/Smad (PubChem assay AID:1347032_TGF_beta_ant), and agonist of the thyrotropin-releasing hormone receptor (PubChem assay AID:1347030_TRHR_ago) in the training dataset; n = 3.

Figure S6

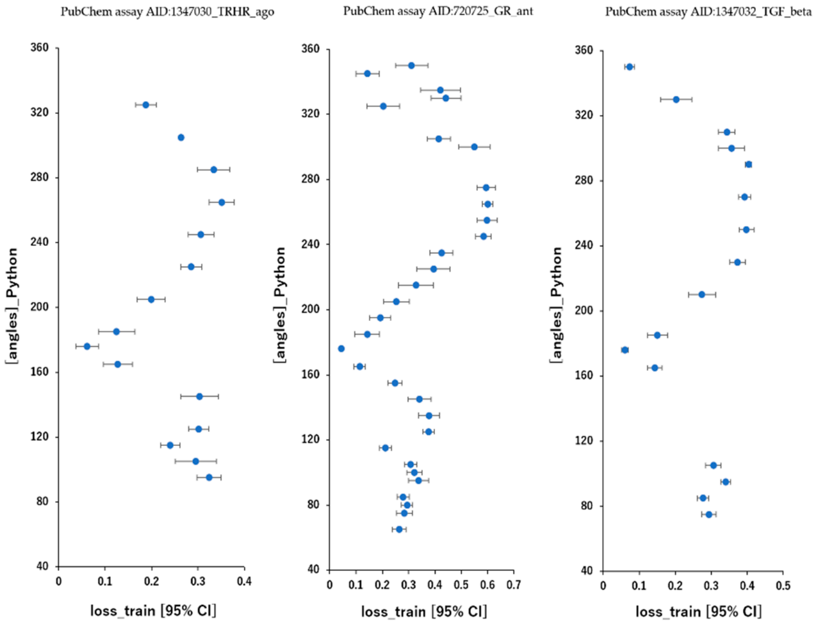

Figure S6. Differences in mean loss levels shown as dots with 95% confident interval (95% CI) as error bars with angles in DeepSnap-DL using TensorFlow and DIGITS of prediction models of the glucocorticoid receptor (PubChem assay AID:720725_GR_ant), TGF-beta/Smad (PubChem assay AID:1347032_TGF_beta_ant), and agonist of the thyrotropin-releasing hormone receptor (PubChem assay AID:1347030_TRHR_ago) in the training dataset; n = 3.

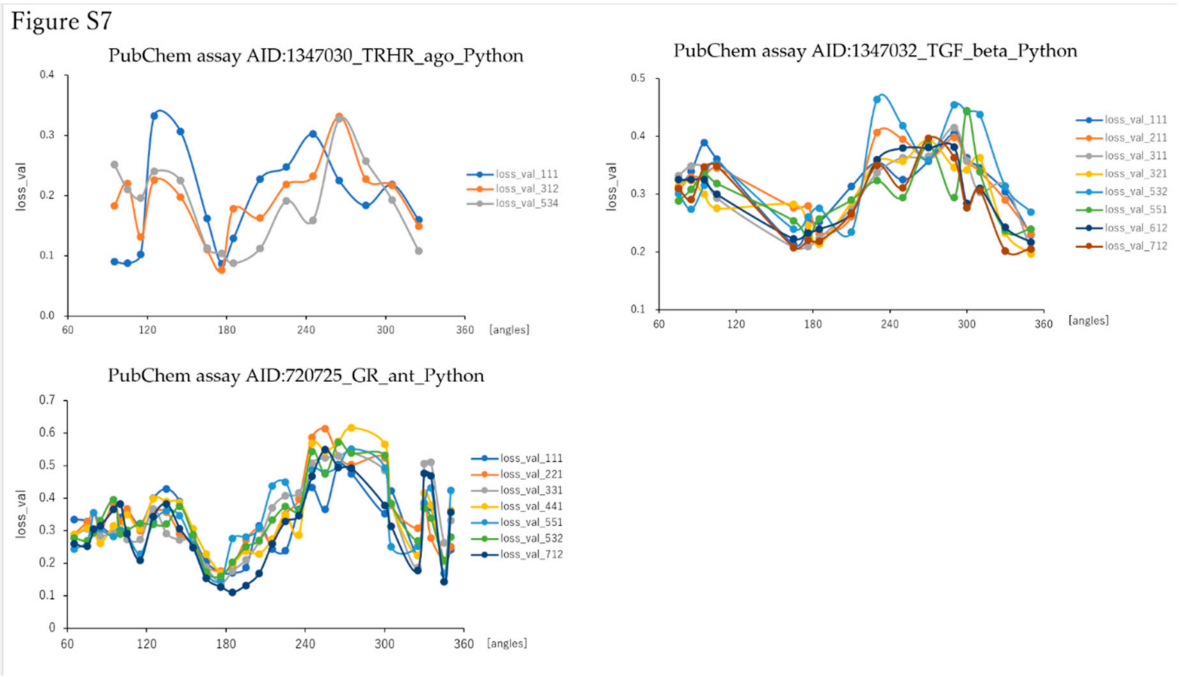

Figure S7. Loss with angles in DeepSnap-DL using TensorFlow and DIGITS of prediction models of the glucocorticoid receptor (PubChem assay AID:720725_GR_ant), TGF-beta/Smad (PubChem assay AID:1347032_TGF_beta_ant), and agonist of the thyrotropin-releasing hormone receptor (PubChem assay AID:1347030_TRHR_ago) in the validation dataset; n = 3.

Figure S8

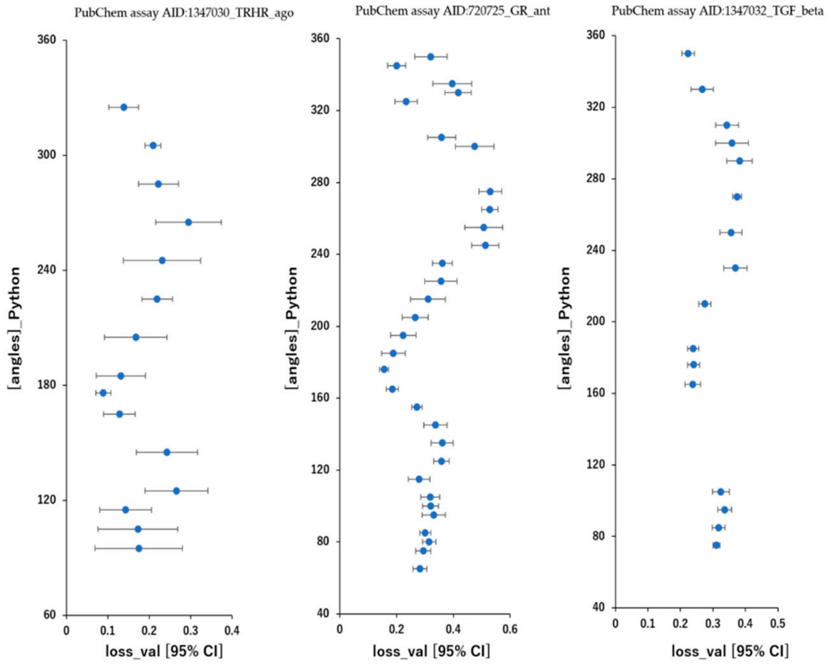

Figure S8. Differences in mean loss levels shown as dots with 95% confident interval (95% CI) as error bars with angles in DeepSnap-DL using TensorFlow and DIGITS of prediction models of the glucocorticoid receptor (PubChem assay AID:720725_GR_ant), TGF-beta/Smad (PubChem assay AID:1347032_TGF_beta_ant), and agonist of the thyrotropin-releasing hormone receptor (PubChem assay AID:1347030_TRHR_ago) in the validation dataset; n = 3.

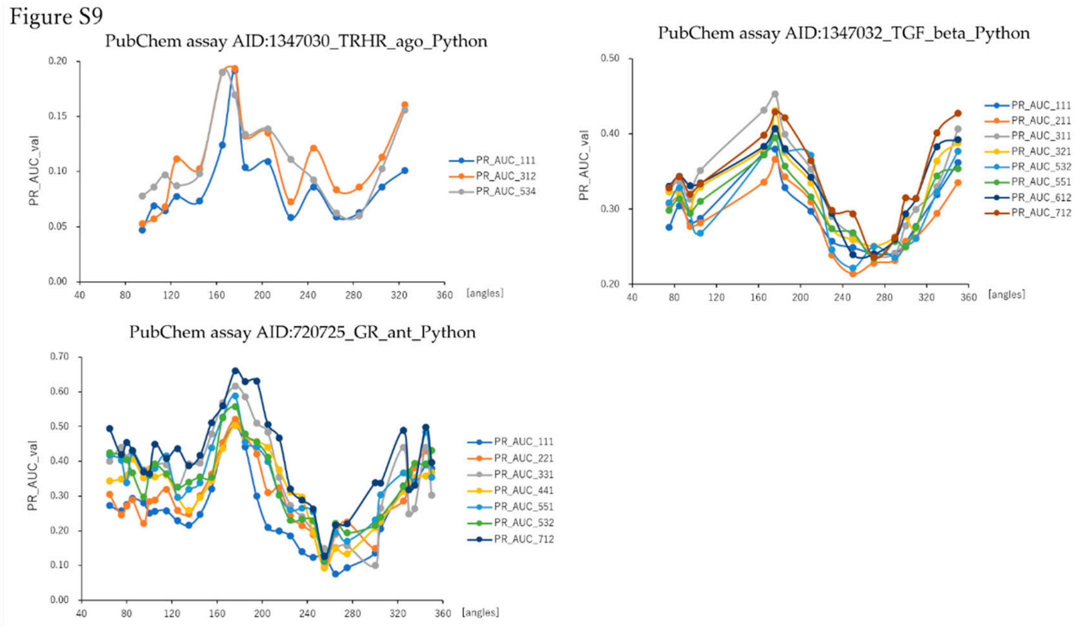

Figure S9. PR_AUC with angles in DeepSnap-DL using TensorFlow and DIGITS of prediction models of the glucocorticoid receptor (PubChem assay AID:720725_GR_ant), TGF-beta/Smad (PubChem assay AID:1347032_TGF_beta_ant), and agonist of the thyrotropin-releasing hormone receptor (PubChem assay AID:1347030_TRHR_ago) in the validation dataset; n = 3.

Figure S10

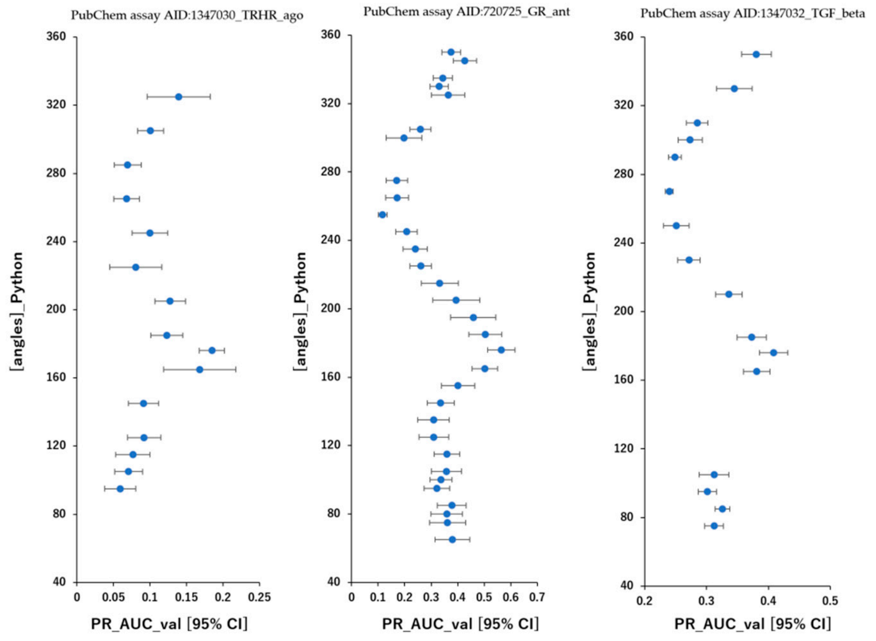

Figure S10. Differences in mean PR_AUC levels shown as dots with 95% confident interval (95% CI) as error bars with angles in DeepSnap-DL using TensorFlow and DIGITS of prediction models of the glucocorticoid receptor (PubChem assay AID:720725_GR_ant), TGF-beta/Smad (PubChem assay AID:1347032_TGF_beta_ant), and agonist of the thyrotropin-releasing hormone receptor (PubChem assay AID:1347030_TRHR_ago) in the validation dataset; n = 3.

Figure S11

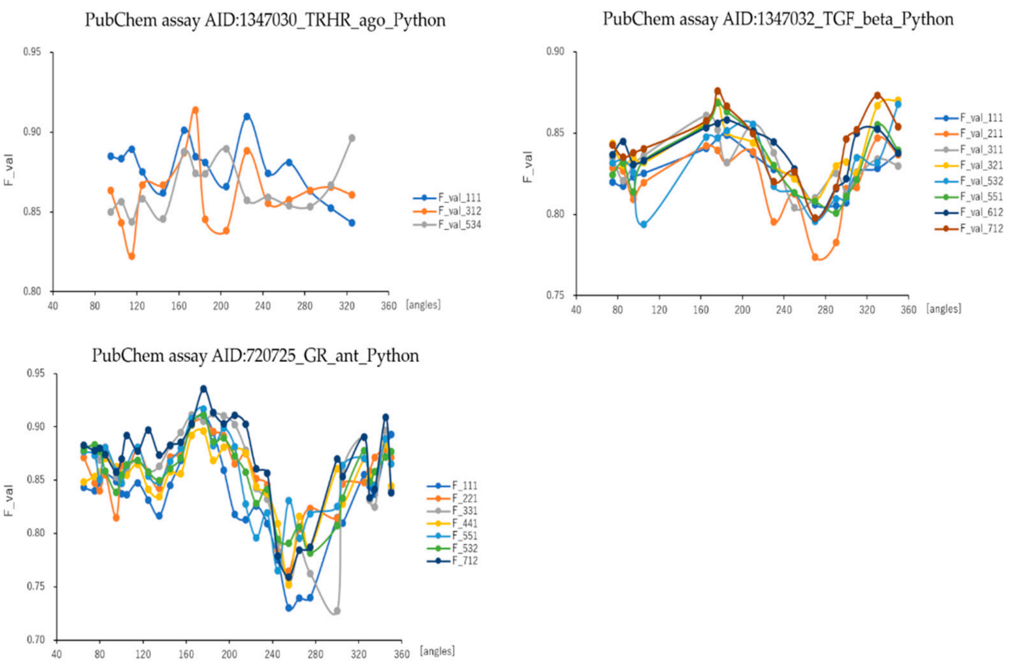

Figure S11. F with angles in DeepSnap-DL using TensorFlow and DIGITS of prediction models of the glucocorticoid receptor (PubChem assay AID:720725_GR_ant), TGF-beta/Smad (PubChem assay AID:1347032_TGF_beta_ant), and agonist of the thyrotropin-releasing hormone receptor (PubChem assay AID:1347030_TRHR_ago) in the validation dataset; n = 3.

Figure S12

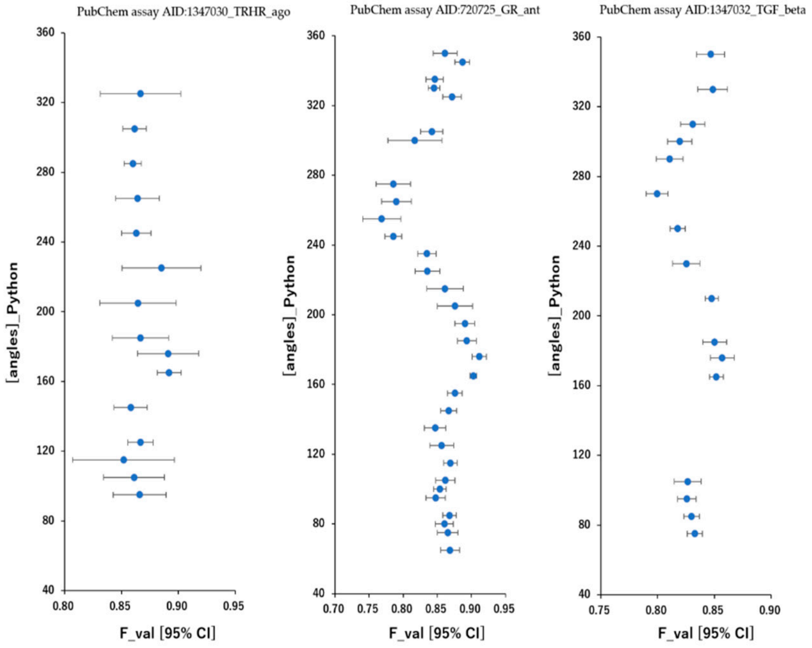

Figure S12. Differences in mean F levels shown as dots with 95% confident interval (95% CI) as error bars with angles in DeepSnap-DL using TensorFlow and DIGITS of prediction models of the glucocorticoid receptor (PubChem assay AID:720725_GR_ant), TGF-beta/Smad (PubChem assay AID:1347032_TGF_beta_ant), and agonist of the thyrotropin-releasing hormone receptor (PubChem assay AID:1347030_TRHR_ago) in the validation dataset; n = 3.

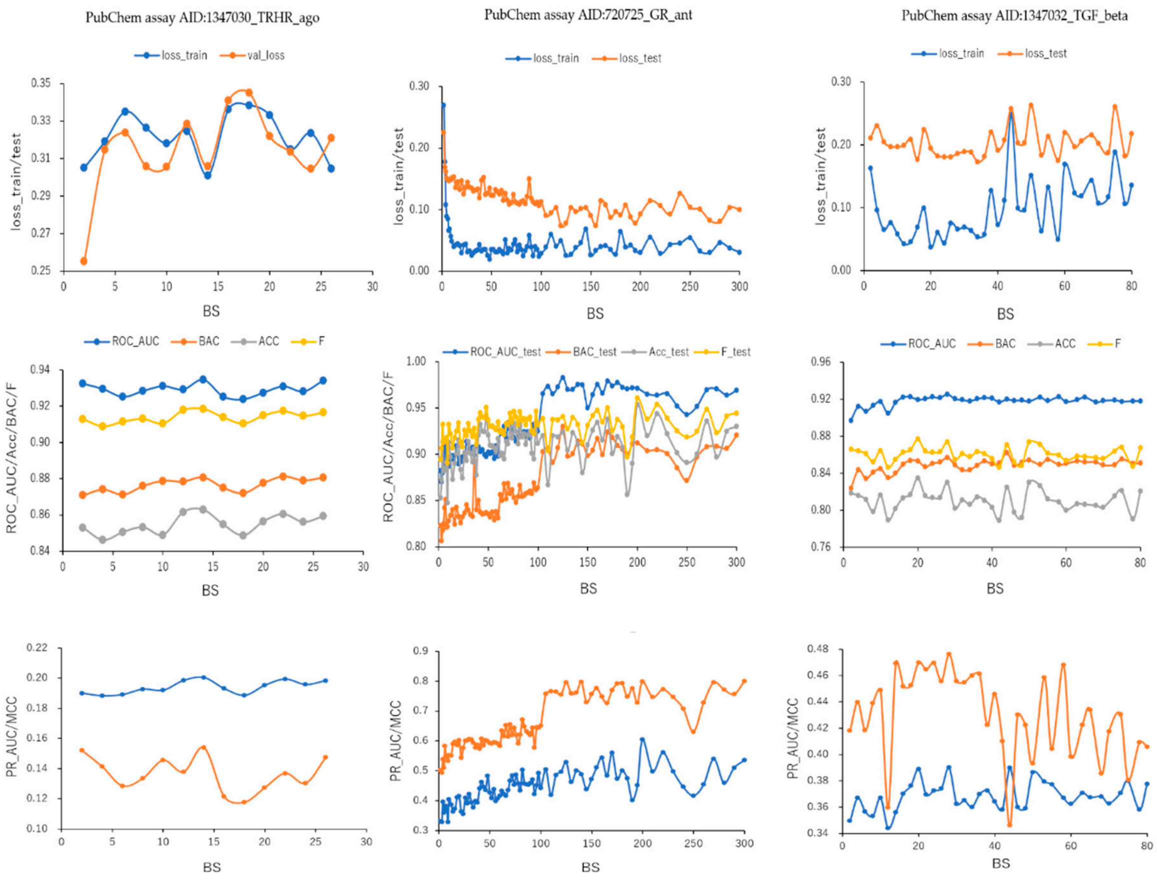

Figure S13. Performance with batch size (BSs) in DeepSnap-DL using TensorFlow of prediction models of the glucocorticoid receptor (PubChem assay AID:720725_GR_ant), TGF-beta/Smad (PubChem assay AID:1347032_TGF_beta_ant), and agonist of the thyrotropin-releasing hormone receptor (PubChem assay AID:1347030_TRHR_ago) in the test dataset; n = 2.
